# Repurposing the anti-malarial drug artesunate as a novel therapeutic agent for metastatic renal cell carcinoma due to its attenuation of tumor growth, metastasis, and angiogenesis

**DOI:** 10.18632/oncotarget.5422

**Published:** 2015-09-28

**Authors:** Da Eun Jeong, Hye Jin Song, Sharon Lim, Se Jeong Lee, Joung Eun Lim, Do-Hyun Nam, Kyeung Min Joo, Byong Chang Jeong, Seong Soo Jeon, Han Yong Choi, Hye Won Lee

**Affiliations:** ^1^ Department of Health Sciences and Technology, SAIHST, Sungkyunkwan University, Seoul, Korea; ^2^ Department of Anatomy and Cell Biology, Samsung Medical Center, Sungkyunkwan University School of Medicine, Seoul, Korea; ^3^ Department of Pathology and Translational Genomics, Samsung Medical Center, Sungkyunkwan University School of Medicine, Seoul, Korea; ^4^ Institute for Refractory Cancer Research, Samsung Medical Center, Sungkyunkwan University School of Medicine, Seoul, Korea; ^5^ Department of Urology, Samsung Medical Center, Sungkyunkwan University School of Medicine, Seoul, Korea; ^6^ Department of Neurogurgery, Samsung Medical Center, Sungkyunkwan University School of Medicine, Seoul, Korea; ^7^ Research Institute for Future Medicine, Samsung Medical Center, Sungkyunkwan University School of Medicine, Seoul, Korea

**Keywords:** renal cell carcinoma, metastasis, artesunate, oncosis, drug repurposing

## Abstract

Despite advances in the development of molecularly targeted therapies, metastatic renal cell carcinoma (RCC) is still incurable. Artesunate (ART), a well-known anti-malarial drug with low toxicity, exhibits highly selective anti-tumor actions against various tumors through generation of cytotoxic carbon-centered free radical in the presence of free iron. However, the therapeutic efficacy of ART against metastatic RCC has not yet been fully elucidated. In the analysis on a dataset from The Cancer Genome Atlas (TCGA) (*n* = 469) and a tissue microarray set from Samsung Medical Center (*n* = 119) from a cohort of patients with clear cell RCC (ccRCC), up-regulation of transferrin receptor 1 (TfR1), which is a well-known predictive marker for ART, was correlated with the presence of distant metastasis and an unfavorable prognosis. Moreover, ART exerted potent selective cytotoxicity against human RCC cell lines (Caki-1, 786-O, and SN12C-GFP-SRLu2) and sensitized these cells to sorafenib *in vitro*, and the extent of ART cytotoxicity correlated with TfR1 expression. ART-mediated growth inhibition of human RCC cell lines was shown to result from the induction of cell cycle arrest at the G2/M phase and oncosis-like cell death. Furthermore, ART inhibited cell clonogenicity and invasion of human RCC cells and anti-angiogenic effects *in vitro* in a dose-dependent manner. Consistent with these *in vitro* data, anti-tumor, anti-metastatic and anti-angiogenic effects of ART were also validated in human 786-O xenografts. Taken together, ART is a promising novel candidate for treating human RCC, either alone or in combination with other therapies.

## INTRODUCTION

Approximately 40% of all patients with renal cell carcinoma (RCC) die of metastasis. Metastases are often present at diagnosis, and relapse after nephrectomy is common [1, 2]. Metastatic RCCs are difficult to remove surgically, are generally resistant to most chemotherapies and radiation, and show only limited sensitivity to cytokine therapy [1, 2]. Although several targeted therapeutics such as vascular endothelial growth factor (VEGF) and mammalian target of rapamycin (mTOR) inhibitors have been approved for the treatment of metastatic disease over the past 10 years, metastatic RCC is still incurable [3].

There is a renewed interest in the discovery of novel anti-cancer drugs from natural compounds and their derivatives [4]. In addition to its established safety and tolerability in anti-malarial treatment, artesunate (ART), a semi-synthetic derivative of the sesquiterpene artemisinin, has shown promise in crossover usage as an anti-cancer agent for a variety of solid tumors [5–10]. Several mechanisms underlying its anti-tumor activities have been proposed, including (1) actions on cell cycle progression [11], (2) disruptions to the intrinsic apoptotic pathway that drive the cell toward a proapoptotic outcome [12, 13], and (3) anti-angiogenic and anti-metastatic properties [14–16]. Recently, oncosis has regained attention since a number of novel anticancer agents have been identified based on their ability to induce oncotic cell death [17]. An important biochemical event leading to oncosis, as opposed to apoptosis, is depletion in intracellular ATP resulting in the impairment of several ionic pumps [18]. Interestingly, ART is a native compound that can induce oncosis in pancreatic [19] and gastric [20] cancer cells.

Although the mechanisms of action of ART and its derivatives have yet to be fully defined, these compounds can exert direct oxidative damage to cancer cells in the presence of free iron by converting themselves into cytotoxic carbon-centered free radicals [21, 22]. Excess iron also promotes oxidative stress via reactive oxygen species (ROS) production [23]. Transferrin receptor 1 (TfR1) has a key role in cellular iron uptake; TfR1 expression is particularly up-regulated in rapidly growing cancer cells, which need excess iron for their rapid proliferation [24]. A number of recent studies have supported the hypothesis that TfR1 is an important biomarker of susceptibility to ART. Specifically, ART has been shown to selectively kill cancer cells [24], and TfR1 expression is significantly correlated with ART sensitivity in various cancer cell lines [25–27].

However, the mechanisms by which ART and its derivatives exert specific anticancer activity against RCC have not yet been defined. This lack of knowledge hinders the development of these compounds in preclinical and clinical settings. Therefore, the aims of this study were two-fold: 1) to investigate the anti-tumor effects of ART on a panel of human RCC cell lines *in vitro* and *in vivo*; and 2) to investigate the underlying mechanisms by which ART exerts its effects, and the clinical implications of ART in metastatic RCC. Since ART derivatives are already in clinical use, we anticipated that our findings would potentially be readily translatable to clinical practice. Our results promise to provide a basis for future development of these compounds as anti-RCC agents, either alone or in rational combination with conventional tyrosine kinase inhibitors (TKIs).

## RESULTS

### TfR1 is up-regulated in clear cell RCC (ccRCC) with distant metastases, and high TfR1 expression correlates with poor prognosis

**Figure 1:**
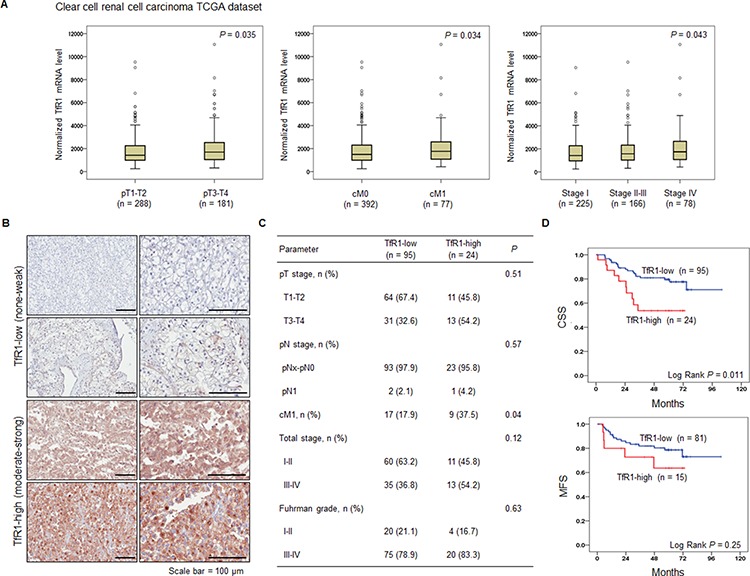
Upregulation of transferrin receptor 1 (TfR1) in human primary renal cell carcinoma (RCC) is correlated with distant metastasis and worse clinical outcomes **A.** Differential mRNA expression of TfR1 in different stages of clear cell RCC (ccRCC). Pathological T stage, clinical M stage, and total stage information was obtained from The Cancer Genome Atlas-Kidney Renal Clear Cell Carcinoma (TCGA-KIRC) RNA sequencing dataset (*n* = 469). The box plots display medians with 95% confidence intervals. **B.** Representative immunohistochemical staining (IHC) of TfR1 from the ccRCC tissue microarray (TMA) cohort. IHC staining of TfR1 was performed and signal intensity was scored as follows: 0, negative; 1, weak; 2, moderate positive; 3, strong positive. Patients were stratified as low (0 or 1) or high (2 or 3). Scale bar, 100 μm. **C.** *P* values for correlations of TfR1 expression with various clinico-pathological characteristics of the TMA cohort. **D.** Kaplan-Meier analysis of cancer-specific survival (CSS) and metastasis-free survival (MFS) according to TfR1 staining intensity in the primary ccRCC TMA cohort. The log-rank test was used to analyze statistical significance.

To determine the clinical relevance and therapeutic implication of ART for patients with metastatic RCC, we examined whether TfR1 expression was correlated with any of the clinico-pathological characteristics of ccRCC. TfR1 mRNA and protein expression levels were obtained from a dataset from The Cancer Genome Atlas (TCGA, http://cancergenome.nih.gov) (*n* = 469) (Figure 1A) and a tissue microarray (TMA) from Samsung Medical Center (Figure 1B–1D), respectively. Using the TCGA mRNA sequencing data, we found that elevated TfR1 expression was correlated with advanced T stage and distant metastasis (Figure 1A). Additionally, stage IV metastatic ccRCCs were found to have significantly higher levels of TfR1 mRNA compared with localized ccRCCs (Figure 1A), suggesting that elevated transcription of TfR1 is significantly associated with locally advanced and metastatic disease states. To further test this hypothesis, we assayed TfR1 expression in 119 surgically removed primary ccRCC tumors on the TMA by immunohistochemistry (IHC) (Figure 1B). Moderate or strong expression of TfR1 (TfR1-high) was detected in 24 out of the 119 (20.2%) tumor tissue samples (Figure 1C). Moreover, TfR1-high tumor tissue was associated with the presence of distant metastasis at diagnosis (Figure 1C). Consistent with this finding, TfR1-high tumor tissue was also correlated with decreased cancer-specific survival (CSS) and metastasis-free survival (MFS) (Figure 1D), indicating that TfR1 could serve as an important prognostic factor for determining patient outcome. Taken together, TfR1 up-regulation is significantly associated with enhanced metastatic potential and worse clinical prognosis of RCC, implying that TfR1 expression data could be used in advance to select patients with RCC for whom ART could be a beneficial therapeutic agent.

### TfR1 in cell proliferation and the invasion of human RCC cells

**Figure 2:**
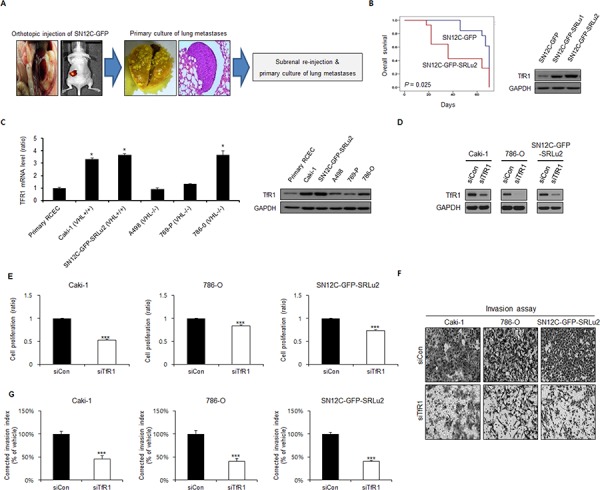
The cell proliferation and invasion Inhibitory effects of TfR1 silencing in human RCC cells **A.** The process of the selection of lung-specific metastatic subpopulations from SN12C-GFP human RCC cells *in vivo*. SN12C cells were injected into the left subrenal capsule space (orthotopic injection), resulting in spontaneous lung metastasis. The subsequent lung-metastatic generations were designated SN12C-GFP-SRLu1 and SN12C-GFP-SRLu2, with SN12C-GFP-SRLu2 cells used in these experiments. **B.** Comparison of parental SN12C-GFP cells versus the subline lung metastatic SN12C-GFP-SRLu2 cells with respect to biological aggressiveness by Kaplan-Meier curves. Immunoblotting was also used to compare the levels of TfR1 between the two cell lines. **C.** Levels of TfR1 mRNA and protein expression in a variety of human RCC cell lines and human renal cortical epithelial cells (RCECs) as measured by qRT-PCR and western blotting. Data are presented as means ± SDs from three independent experiments. *P < 0.05. **D–G.** To examine whether TfR1 is involved in cell proliferation and invasion in human RCC, Caki-1, 786-O and SN12C-GFP-SRLu2 cells were transfected with non-targeting siRNA (siCon) or TfR1 siRNA (siTfR1) for 48 h. The level of cyclin D1 was determined by western blot (D), and cell growth and invasive activity were assessed using a Ezy-Cytox viability assay (E) and a Transwell invasion assay (F–G), respectively *in vitro*. Data are presented as means ± standard deviations (SDs) from three independent experiments. ***P < 0.05.

RCC metastases to the lungs are the most frequent type of metastasis, with prevalence rates as high as 72% and 76% in autopsy studies [28]. To recapitulate metastatic RCC *in vitro*, we established an RCC cell line, SN12C-GFP-SRLu2, which is capable of efficiently colonizing the lungs in mice. This cell line is a lung metastatic subline that was derived from SN12C cells expressing green fluorescent protein (SN12C-GFP) (Figure 2A). Consistent with our clinical findings, SN12C-GFP-SRLu2 cells displayed increased TfR1 expression (Figure 2B) and decreased overall survival relative to their parental counterparts (Figure 2B) due to enhanced tumorigenic and metastatic potential.

To investigate the value of TfR1 as a predictive marker of the suitability of ART for the treatment of RCC, we next examined TfR1 expression levels in various human RCC cell lines [VHL wild-type RCC cell lines = Caki-1 and SN12C-GFP-SRLu2; VHL-deficient (mutated or hypermethylated) RCC cell lines = A498, 769-P, and 786-O] and normal renal cortical epithelial cells (RCECs) (Figure 2C). The mRNA and protein levels of TfR1 were higher in Caki-1, 786-O, and SN12C-GFP-SRLu2 cells than in A498 and 769-P RCC cells, and were also much higher than in RCECs (Figure 2C). This finding is consistent with the observed up-regulation of TfR1 in cancer cells.

To verify the role of TfR1 in the progression of RCC, the effect of small interfering RNA (siRNA) mediated gene silencing of TfR1 on tumor cell growth and invasion activity of human RCC cells were analyzed *in vitro*. TfR1 silencing with siRNA in Caki-1, 786-O and SN12C-GFP-SRLu2 cells (Figure 2D) significantly diminished cell proliferation (Figure 2E) and the invasiveness (Figure 2F and 2G), demonstrating that increased expression of TfR1 is involved in gain of tumorigenic and metastatic potential of RCC cells.

### *In vitro* selective cytotoxicity and sensitizing effect to sorafenib of ART in human RCC cells

**Figure 3:**
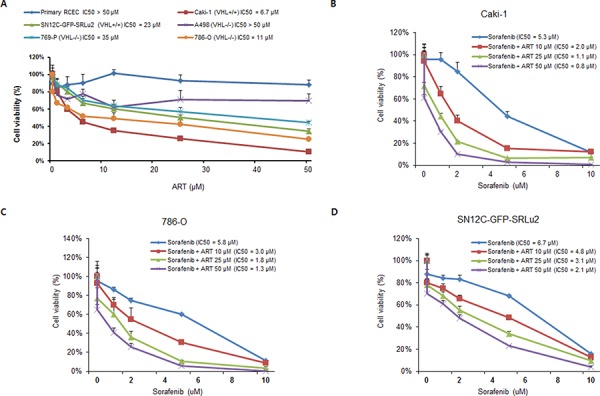
Artesunate (ART) decreases the viability of human RCC cells *in vitro* and enhances the susceptibility of RCC cells to sorafenib **A.** Cytotoxic effects of ART on human RCC cells. RCC cells and normal renal epithelial cells (RCECs) were seeded into 96-well plates and treated with the indicated concentrations of ART for 72 hours. The mean values from three independent Ezy-Cytox viability assays were plotted and the half maximal inhibitory concentration (IC50) values for ART with respect to cell growth inhibition were calculated. Values are expressed as means ± SDs. **B–D.** Effect of combination treatment with sorafenib plus ARon the viability of human RCC cells. Caki-1, 786-O, and SN12C-GFP-SRLu2 cells were treated for 72 hours with different concentrations of sorafenib in combination with 10 μM, 25 μM and 50 μM ART. The mean values from three independent Ezy-Cytox viability assays were plotted and IC50 values for sorafenib with respect to cell growth inhibition were calculated. Values are expressed as means ± SDs.

In subsequent *in vitro* studies using a human RCC preclinical platform to determine the therapeutic potential of ART, ART exhibited remarkably selective, dose-dependent cytotoxicity in the micromolar range against RCC cell lines with elevated levels of TfR1 (Caki-1, 786-O, and SN12C-GFP-SRLu2) (Figure 3A), strengthening the potential of TfR1 as a companion diagnostic biomarker for ART in RCC. Since TfR1 expression and ART-mediated suppression of cell growth were most highly correlated in Caki-1, 786-O, and SN12C-GFP-SRLu2 cells, these cell lines were selected for further preclinical tests of the anti-tumor activities of ART.

In the clinical trials that have been conducted thus far, therapies that target single agents have not yielded a substantial clinical benefit against metastatic RCC [29]. No established consensus has yet been reached regarding the best treatment approach for patients with RCC that has acquired resistance to conventional therapies. Although few studies have explored the value of ART as a combination partner in treatment regimens, ART has been used in combination with other agents, chosen based on target diversity, that mutually support each other [30]. Thus, we next explored the activity of ART alone and in combination with clinically approved target agents for metastatic RCC (sunitinib, sorafenib, and everolimus). To determine the extent of synergy between ART and these agents, Caki-1, 786-O, and SN12C-GFP-SRLu2 cells were exposed for 72 hours to different concentrations of each agent, both in the presence and absence of ART. Among these agents, only sorafenib exerted significant synergistic inhibition with ART against RCC cell lines (Figure 3B–3D). Although the underlying mechanism driving the synergism between ART and sorafenib needs to be further elucidated, these results suggest that ART may have potential therapeutic value as a sensitizer of standard strategies for the treatment of patients with advanced RCC.

### ART induces G2/M cell cycle arrest, but not apoptosis, in human RCC cells

**Figure 4:**
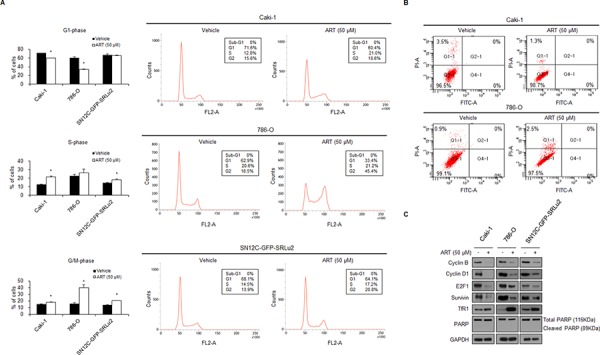
ART-treated RCC cells accumulate in G2/M phase, but ART does not induce apoptosis in Caki-1, 786-O, or SN12C-GFP-SRLu2 cells **A.** Caki-1, 786-O, and SN12C-GFP-SRLu2 cells were treated with 50 μM ART or vehicle control for 48 hours. Cell cycle distribution was then analyzed by flow cytometry after propidium iodide (PI) staining. The left panels summarize the flow cytometry results for the different cell cycle stages. Data are expressed as means ± SDs from 3 independent experiments. *P < 0.05. The right panels show representative examples of cell cycle analysis by flow cytometry. Cell subpopulations are expressed as percentages of the total population. **B.** Caki-1, 786-O, and SN12C-GFP-SRLu2 cells were treated with 50 μM ART or vehicle control for 48 hours and then stained with Annexin V and PI. Representative FACS cytograms of stained cells are shown. Viable cells (Annexin V and PI negative) are in the lower left quadrant. Early apoptotic cells (Annexin V positive/PI negative) are in the lower right quadrant. Terminal apoptotic/necrotic cells (Annexin V positive/PI positive) are in the upper right quadrant. Cell subpopulations are expressed as percentages of the total population. **C.** Caki-1, 786-O, and SN12C-GFP-SRLu2 cells were cultured with DMSO and ART (50 μM) for 72 hours, after which whole cell extracts were generated and subjected to SDS-PAGE. Western blotting was then performed using the indicated antibodies.

Since treatment with ART significantly suppressed the growth of RCC cells, we next investigated the mechanism of this suppression by determining whether ART inhibits cell cycle progression or induces apoptotic cell death. Flow cytometry analysis showed that treatment with ART changed the cell cycle stage distribution in Caki-1, 786-O, and SN12C-GFP-SRLu2 cells. Specifically, these cells were arrested in the G2/M phase at 48 hours after ART treatment (Figure 4A). Treatment with ART increased the fractions of cells in G2/M, while the proportions of cells in S phase were either slightly increased (Caki-1, SN12C-SRLu2) or unchanged (786-O) (Figure 4A). These findings are consistent with partial arrest at the G2/M phase of the cell cycle. Consistent with the flow cytometry results, decreased protein levels of cyclin B, cyclin D1, and E2F1 were observed (Figure 4C). Since these proteins regulate cell cycle progression, this finding supports the idea that ART treatment leads to G2/M cell cycle arrest. Since cyclin D1 regulates G1 phase transition, the observed decrease in cyclin D1 levels may be secondary to the G2/M arrest caused by ART treatment.

Interestingly, at day 2, cell cycle analysis revealed that treatment of the three RCC cell lines with 50 μM ART did not generate a sub-G1 fraction (Figure 4A). This finding indicates that ART does not induce apoptosis in human RCC cells. As a complementary technique to study the relationship between ART and apoptosis, RCC cells were cultivated in the presence of ART (50 μM) for 48 hours and then double-stained with Annexin V-FITC/propidium iodide (PI). As shown in Figure 4B, apoptotic cells were rarely found in any cell line after treatment with 50 μM ART. Moreover, poly (ADP-ribose) polymerase (PARP) cleavage was not significantly increased as assessed by Western blot analysis (Figure 4C).

### ART-induced cell death is accompanied by ultrastructural features typical of oncosis, which occurs via ROS generation and ATP depletion, in RCC cells

**Figure 5:**
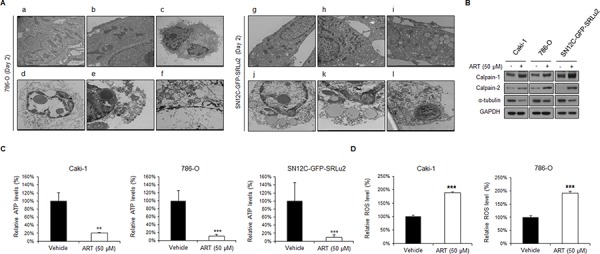
ART mediates oncotic cell death in human RCC cells via reactive oxygen species (ROS) generation and intracellular ATP depletion **A.** (a-b) Untreated control and (c-f) ART-treated 786-O cells; (g–h) untreated control and (i–l) ART-treated SN12C-GFP-SRLu2 cells. High-magnification electron micrograph showing disruption of nuclear and cytoplasmic organization, cytoplasmic swelling, dilation of ER elements, swollen mitochondria, and plasma membrane rupture following ART treatment in 786-O and SN12C-GFP-SRLu2 cells. **B.** Effects of ART on the expression of calpain-1, calpain-2 and α-tubulin in human RCC cells. Caki-1, 786-O, and SN12C-SRLu2 cells were treated with ART (50 μM) for 6 hours. The levels of calpain-1, calpain-2 and α-tubulin were then analyzed by immunoblotting with the indicated antibodies. **C.** Treatment with ART decreases the level of intracellular ATP and leads to oncosis. Intracellular ATP levels were determined by measuring the activity of luciferase on its substrate, D-luciferin. Caki-1, 786-O, and SN12C-SRLu2 cells were treated with DMSO or ART (50 μM) for 48 hours and lysed. The resultant luminescence of each well was measured with a luminometer. **, *P* < 0.01; ***P < 0.001 **D.** Relative ROS production in Caki-1 and 786-O cells incubated with or without 50 μM ART for 24 hours. Data are expressed as means ± SDs from 3 independent experiments. Closed bar: DMSO control; open bar: ART (50 μM). ***P < 0.001.

Since ART did not induce apoptosis in RCC cells, we next applied the gold standard technique for identifying oncosis, electron microscopy, to examine ART-treated cells on the ultra-structural level. The control 786-O (Figure 5Aa–5Ab) and SN12C-GFP-SRLu2 (Figure 5Ag–5Ah) cells exhibited normal cellular morphology with intact nuclei and other organelles. In contrast, high-magnification electron micrographs of ART-treated 786-O (Figure 5Ac–5Af) and SN12C-SRLu2 (Figure 5Ai–5Al) cells revealed dilated nuclei, irregular clumping of chromatin, cytoplasmic swelling, severely damaged and swollen organelles, numerous cytoplasmic vacuoles, and ruptures in the plasma membrane. These morphologic characteristics are consistent with oncosis-like cell death [31] and support our hypothesis that ART induces oncosis, rather than apoptosis, in RCC cells.

To investigate the mechanism by which ART induces oncosis, we analyzed the expression of oncosis-associated proteins in RCC cells by western blotting. Calpains are believed to be involved in oncosis-like cell death [32]. Calpains comprise a family of calcium-dependent cysteine proteases that perform limited proteolytic cleavage on a variety of cellular substrates, including cytoskeletal proteins [33]. In addition, since ultra-structural examination of ART-treated cells revealed a fuzzy morphology, we hypothesized that the cytoskeleton might also be affected. Oncosis has been shown to be triggered by ATP depletion and ATP depletion can cause actin depolymerization, breakdown of the actomyosin network, changes in the lipid order of the plasma membrane, and membrane blebbing. As shown in Figure 5B, ART-treated cells exhibited significantly increased calpain-1 and calpain-2 expression and decreased α-tubulin compared with control cells.

ATP depletion in oncosis affects ion pumps, leading to the collapse of mitochondrial potential [32]. Specifically, the cytomembrane is destroyed due to the inactivation of ion pumps, which is caused by ATP depletion. In parallel with these changes, ROS also accumulate and are commonly used as markers of cellular damage. Although ROS generation and mitochondrial membrane changes are key events in both apoptosis and necrosis, ATP depletion is only observed in oncosis. Interestingly, ART-treated Caki-1 and 786-O cells exhibited decreased intracellular ATP concentrations compared with control cells (Figure 5C). In addition, ART-treated cells tended to accumulate ROS, which are an indicator of mitochondrial damage (Figure 5D). These data suggest that ART is a potent cytotoxic drug that displays an unusual mode of action, i.e., triggering oncosis.

### Inhibitory effects of ART on clonogenicity and invasion properties of human RCC cells

**Figure 6:**
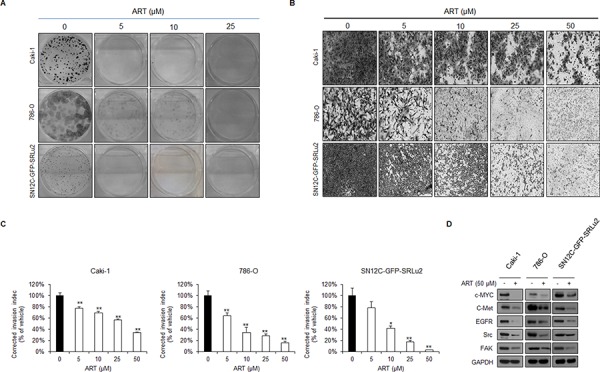
ART inhibits the colony forming and invasion abilities of human RCC cells in a dose dependent manner **A.** Evaluation of the effect of ART treatment on clonogenicity in Caki-1, 786-O, and SN12C-GFP-SRLu2 cells. Representative images of Caki-1, 786-O, and SN12C-GFP-SRLu2 cells stained for focus formation after 2 weeks of DMSO or ART treatment (5, 10 or 25 μM). **B–C.** Invasion of Caki-1, 786-O, and SN12C-GFP-SRLu2 cells through Boyden transwell chambers was determined after treatment with DMSO or ART (5, 10, 25 or 50 μM). Representative images of the bottom surface are shown. Averages of ten random microscopic fields are presented for each condition. Data are expressed as means ± SDs from 3 independent experiments. *P < 0.05; **P < 0.01. **D.** Caki-1, 786-O, and SN12C-GFP-SRLu2 cells were treated with or without ART (50 μM) for 72 hours, and western blotting was then performed using the indicated antibodies.

We next performed focus forming assays (Figure 6A) and transwell invasion assays (Figure 6B) to examine other properties that are required for distant metastasis of RCC, specifically, clonogenicity and invasiveness. These experiments revealed that ART significantly diminished the clonogenicity (Figure 6A) and invasiveness (Figure 6B) of human RCC cells. Especially, foci formation was completely abolished in Caki-1 and SN12C-GFP-SRLu2 cells in the presence of 5 μM ART while ART demonstrated suppression of foci formation of 786-O cells in a dose dependent manner (Figure 6A). Similarly, the addition of the ART to these cells dose-dependently reduced their capacity to invade (Figure 6B). In support of these findings, treatment with ART down-regulated the expression of multiple molecules associated with maintaining cancer stemness and metastatic potential, including c-MYC, epidermal growth factor receptor (EGFR), c-MET, Src, and focal adhesion kinase (FAK) (Figure 6C). These results strengthen the potential therapeutic value of ART in the treatment of metastatic RCC.

### ART inhibits the proliferation, migration, and formation of capillary structures in human umbilical vein endothelial cells (HUVECs), in addition to inhibiting vascular endothelial growth factor receptor 2 (VEGFR2) signaling *in vitro*

**Figure 7:**
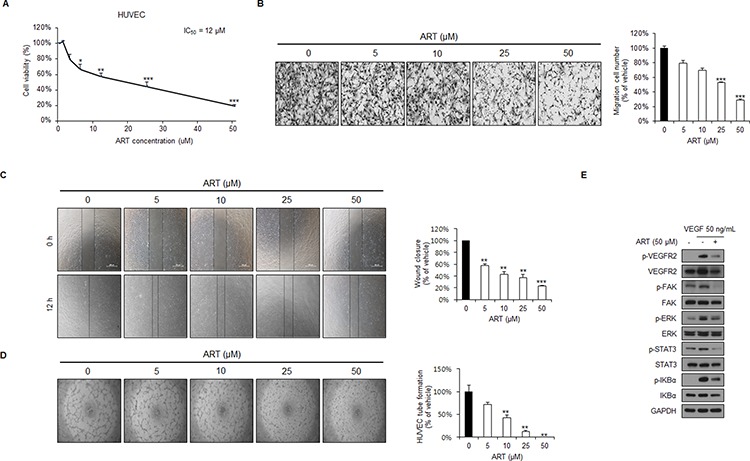
ART inhibits cell proliferation, migration, and capillary structure formation of human umbilical vein endothelial cell (HUVECs), in addition to inhibiting VEGFR2 signaling *in vitro* **A.** ART inhibits the proliferation of HUVECs as analyzed by the Ezy-Cytox assay. Results were normalized to DMSO controls. Data are expressed as means ± SDs from 3 independent experiments. *P < 0.05; **P < 0.01; ***P < 0.001 **B.** ART inhibits HUVEC migration in a transwell assay. A total of 2 × 10^4^ HUVECs were seeded in the top chamber and treated with either DMSO or 5, 10, 25 or 50 μM ART. After 6 hours, migrated HUVECs were stained and quantified. Data are expressed as means ± SDs from 3 independent experiments. ***P < 0.001. **C.** ART inhibits HUVEC migration in the wound healing assay. HUVECs were grown to full confluence in 12-well plates, wounded with a pipette tip, and then treated with DMSO or 5, 10, 25 or 50 μM ART. **P < 0.01; ***P < 0.001. **D.** ART inhibits tube formation of HUVECs. After pretreatment with DMSO or 5, 10, 25 or 50 μM ART for 12 hours, HUVECs were seeded on a Matrigel layer and then further treated with DMSO or 5, 10, 25 or 50 μM ART for 6 hours. The numbers of tubular structures in each group were counted manually; data are expressed as percent inhibition, with the value of vehicle-treated cells set to 100%. **P < 0.01 **E.** ART inhibits VEGFR2 downstream signaling in HUVECs. GAPDH expression was used as an internal control.

Tumor angiogenesis is the rate-limiting step in tumor progression and provides both oxygen and nutrients for tumor growth, invasion, and metastasis [34, 35]. We next investigated the effects of ART on endothelial cell lines *in vitro* and found that ART exerted a profound anti-proliferation effect on HUVECs in a dose-dependent manner (Figure 7A). In addition, transwell migration (Figure 7B) and wound healing assays (Figure 7C) revealed that ART dose-dependently inhibited the migration of HUVECs. Moreover, while vehicle-treated HUVECs plated on a Matrigel surface formed capillary-like structures within six hours, tubule formation was significantly suppressed in ART-treated HUVECs in a dose-dependent manner (Figure 7D).

We next examined the mechanism underlying ART-mediated angiogenesis. Vascular endothelial growth factor receptor 2 (VEGFR2) regulates endothelial cell proliferation, and tube formation [36]. VEGF binds to specific transmembrane receptors on endothelial cells, which results in the phosphorylation of various downstream signal transduction molecules [35, 37]. ART significantly suppressed VEGF-induced phosphorylation of VEGFR2 and its downstream targets in HUVECs (Figure 7E). These results indicate that ART exerts its anti-angiogenic effects by directly targeting VEGFR2 on the surface of endothelial cells and further antagonizing the VEGFR2-mediated downstream signaling cascade, indicating that ART may be well-tolerated and could potentially be used as an anti-angiogenic agent in RCC.

### ART inhibits tumor growth, metastasis and tumor angiogenesis in 786-O xenografts *in vivo*

**Figure 8:**
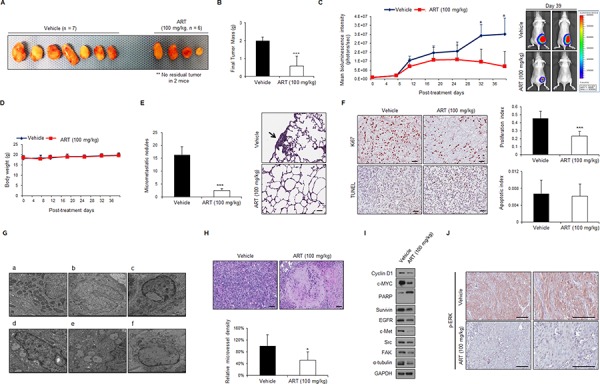
ART exerts anti-tumor, anti-metastasis and anti-angiogenesis effects *in vivo* against 786-O subcutaneous xenografts **A–B.** Images and average tumor weights of subcutaneously implanted 786-O-Luc human RCC tumors at the end of *in vivo* experiment. Tumor-bearing mice were treated intraperitoneally with ART at 100 mg/kg (*n* = 6) or vehicle (*n* = 7) daily after the 786-O-Luc cell tumors (delivered subcutaneously in the flank) reached an average volume of 100 mm^3^. Treatment with ART resulted in significantly reduced tumor growth versus the vehicle control. Moreover, the implanted tumor had disappeared by the end of the experiment in 2 of the 6 mice treated with 100 mg/kg ART. ***P < 0.001. **C.** Bioluminescence imaging of 786-O subcutaneous tumor xenografts and body weight changes in ART-treated and vehicle-treated mice. Tumor growth was assessed by quantifying the bioluminescence signal in each mouse; error bars represent SEMs. Images of representative mice in the vehicle-treated (top) and ART-treated (bottom) groups are shown. *P < 0.01 **D.** Body weight of mice treated with vehicle or ART (100 mg/kg). Data are represented as the means ± SD. **E.** Comparison of numbers of pulmonary micro-metastatic nodules between un-treated (*n* = 10) and ART-treated mice (*n* = 10). After 3 weeks of treatment, the mice were killed, and the metastatic nodules of lungs were counted. Data are presented as the mean ± SDs. ***P < 0.001. Black arrow, micrometastatic nodule in lung. **F.** Cell proliferation and apoptosis were measured immunohistochemically by performing Ki-67 and TUNEL staining. Scale bar, 100 μm. **G.** Typical ultrastructural characteristics of oncotic cell death induced by ART treatment (c–f) were confirmed by electron microscopy examination of harvested tumor tissue. (a–b) Vehicle control. **H.** Anti-angiogenic effects of ART in RCC xenografts were validated by increased amount of tumor necrosis (upper panel) and reduced microvessel density (MVD) (lower panel). Frozen sections of 786-O tumor tissue were analyzed by IHC using anti-CD31 antibodies. The tumor MVD was quantified for each condition at the end of ART treatment. *P < 0.05 **I.** Western blot analysis of samples from each group of mice was performed with the indicated antibodies. **J.** IHC analysis of removed tumor tissue using anti-p-ERK1/2 antibodies. Scale bar, 100 μm.

To investigate the effect of ART *in vivo*, mice bearing subcutaneous xenografts of ccRCC cells (786-O-Luc cells, luciferase tagged for *in vivo* bioluminescence imaging) were treated daily intraperitoneally with ART (100 mg/kg) or vehicle alone as a control. As shown in Figure 8A, ART treatment significantly suppressed tumor growth (*n* = 4) or induced tumor regression (*n* = 2) for the 786-O xenografts compared with vehicle alone. As shown in Figure 8B, tumor weight of ART-treated mice was remarkably lower than that of control group, and ART strongly inhibited tumor growth (Figure 8C) with good *in vivo* tolerability (Figure 8D). Furthermore, 1 × 10^6^ of 786-O-Luc cells were i.v. injected into lateral tail vein of nude mice to examine the effects of ART (100 mg/kg) on the metastatic activity of cancer cells. ART demonstrated meaningful reduction of microscopic lung metastatic foci (Figure 8E). These results indicate that ART inhibits the ability of tumor growth and pulmonary metastasis of RCC cells significantly.

To better understand the mechanism by which ART exerts its anti-tumor and anti-metastatic activities *in vivo*, tumor tissue was isolated from 786-O xenografts at the end of the treatment course and analyzed by IHC. We observed that the ART-treated xenograft tumors contained significantly decreased levels of Ki-67 (Figure 8F), indicating that these tumors contained fewer proliferating cells than control-treated tumors. We next investigated whether ART induced apoptosis in 786-O xenograft tumors by terminal deoxynucleotidyl transferase (dUTP) nick end labeling (TUNEL) staining. These experiments revealed that ART did not increase the amount of apoptotic cells in the tumors, a finding that is consistent with the *in vitro* results (Figure 8F). Importantly, TUNEL-positive cells were counted only in regions of the intact tumor in which the central necrosis that is typically observed in xenografts did not interfere with the quantification of apoptotic cells.

Alternative mechanisms, such as the induction of oncotic cell death and/or the inhibition of angiogenesis, appear to be responsible for the antitumor effects of ART. Electron microscopy analyses demonstrated striking morphological features of ART-treated cells (Figure 8G). Specifically, these cells exhibited perturbed cytoarchitecture with numerous cytoplasmic vacuoles, disintegrated plasma membranes, swollen nuclei, and internally disorganized mitochondria (Figure 8G). Additionally, quantification of the tumor mean vessel density (MVD) at the end of ART treatment revealed that 786-O tumor vasculature was significantly decreased by ART treatment at 100 mg/kg (Figure 8H). Moreover, this decrease was correlated with a significant increase in necrosis, as evidenced by the extensive necrotic areas in ART-treated cells (Figure 8H). This inhibition of angiogenesis correlated with increased oncotic death of tumor cells and increased central necrosis. Finally, downregulation of cyclin D1, c-MYC, survivin, c-Met, EGFR, Src, FAK, and α-tubulin expression was observed in 786-O tumor xenografts treated with ART (Figure 8I). We also observed a significant change in ERK pathway activation in the 100 mg/kg ART-treated group at this time (Figure 8J). Taken together, these data demonstrate that ART exerts multiple anti-tumor and anti-metastatic effects on cell proliferation, oncotic cell death, and tumor angiogenesis *in vivo*. Additionally, ART is also known to have a good safety profile. Thus, ART may be a promising anticancer drug candidate for treating RCC.

## DISCUSSION

Until the past decade, the treatment options for patients with metastatic RCC were extremely limited. As an alternative approach, some pharmaceutical companies have adopted a drug repurposing approach to accelerate the drug discovery and development process. Drug repurposing, which is the process of identifying new or different therapeutically useful indications for existing marketed drugs by targeting alternative disorders, has already been proven to effectively address unmet medical needs for cancer [38–42]. For example, several classes of Food and Drug Administration (FDA)-approved mitochondrially-targeted antibiotics can be repurposed across multiple tumor types based on the ability to eradicate cancer stem cells [40].

Since many effective drugs derived from medical herbs possess diverse pharmacological activities, they may serve as valuable resources for procuring novel anti-tumor compounds. Artemisinin and its analogues possess anti-malarial activity in addition to a rich assortment of anti-cancer activities [9]. The hemisuccinate group in ART confers substantial water solubility and high oral bioavailability, resulting in a favorable pharmacological profile [43]. As a new class of anticancer drugs, ARTs have many advantages including low toxicity to normal tissue/cells [44], low cross-drug resistance [5], and synergistic effects with many traditional chemotherapeutic anticancer drugs [6]. However, the therapeutic efficacy and mode of action of ART in human RCC cells are poorly understood.

Here we characterized several key features of ART action in human RCC cells at the cellular and molecular levels. Our results provide a foundation for future in-depth studies of the effects of ART on additional types of cancer cells. In the present investigation, we reported that ART exerts anti-tumorigenic and anti-metastatic effects in RCC preclinical models and that ART also has anti-angiogenic effects on HUVEC cells. ART has been shown to inhibit tumor growth; induce growth cycle arrest; promote oncosis; inhibit tumor angiogenesis, clonogenicity, and tissue invasion; and inhibit cancer metastasis in human RCC cells without significant toxicity. In addition, we found that ART potentiates the anti-tumor effects of sorafenib in RCC. To the best of our knowledge, this is the first study demonstrating that ART has potential as an anti-tumor agent for metastatic RCC, and also identifies TfR1 as a potential marker for predicting the effect of ART on RCC cells.

The incorporation of high-quality biomarkers in the early development of novel agents is important for guiding later development. ART possesses a unique anti-cancer activity via an iron-dependent mechanism. This mechanism is particularly interesting because TfR1 has been shown to be up-regulated in locally advanced and metastatic RCC. ART internalization into cancer cells has been shown to depend on TfR1 expression [25]. Moreover, the oxidative damage caused by ROS and reactive nitrogen species is controlled by cellular iron homeostasis [45], implying a potential link between oxidative stress and TfR1-mediated iron uptake. Recent studies also have reported that ROS induce the expression of TfR1 by activating p38-MAP kinase [46], thus facilitating iron uptake and the Fenton reaction, which consequently results in more toxic ROS. However, oxidative damage alone is not sufficient to explain all the anticancer activities of ART [47, 48]. In one study, dihydroartemisinin was shown to downregulate the level of surface TfR1 by inducing TfR1 palmitoylation and an unexpected lipid raft-mediated internalization pathway. This internalization led to a decrease in TfR1-mediated iron uptake, which may be one ROS-independent anticancer mechanism of ART [9].

Our results show that ART induces a form of oncosis-like cell death, which may or may not be accompanied by apoptosis consistent previous reports [19, 20]. ART-treated RCC cells showed typical morphologic features of oncosis rather than apoptosis. These features include ATP depletion, ROS generation, and calpain activation. Previous studies have indicated that calpain cleaves its target molecules, which include spectrin, paxillin, and vinculin, thereby inducing an oncotic phenotype [32]. ATP depletion below a certain threshold may direct the cell death pathway towards oncosis [49, 50]. One study found that severe depletion of ATP in mouse renal proximal tubule cells resulted in uniform oncosis-mediated cell death [51]. However, under certain stress conditions neither oncosis nor apoptosis is dominant, suggesting that the two processes can occur simultaneously and may also exhibit cross-talk [52]. Therefore, morphological changes are not the most appropriate endpoint measurement of cell death, which restricts the usefulness of monitoring morphological changes alone in clinical studies.

Artemisinin derivatives can elicit broad-spectrum modulatory effects on a wide panel of signaling molecules and pathways [27]. However, the exact signaling molecules and other targets that interact directly with artemisinin derivatives vary in different cell lines. Thus, the precise activities of ART are likely to be context-dependent. The significant down-regulation of cyclin B, cyclin D1, E2F1, survivin, and c-MYC in human RCC cells mediated by ART may be sufficient to arrest cell cycle progression and to suppress cancer stemness [53–59]. More importantly, this is the first demonstration that ART can decrease the levels of EGFR, c-MET, Src, and FAK in a human RCC cell line. In ccRCC, EGFR overexpression has been shown to be associated with rapid tumor growth and a high tumor grade [60]. Another recent study demonstrated that ART activity in cancer cells is mediated by signaling pathways downstream of EGFR [61]. In various malignancies, dysregulation of c-Met and Src has been shown to regulate invasion and metastasis and correlates with poor patient survival in metastatic RCC [62–65]. Specifically, the primary target of ART in RCC, the precise molecular mechanisms by which ART exerts cytoplasmic organelle damage, and the basis for its preferential effect on RCC cells all remain to be elucidated.

The diversity of ART targets suggests that ART is ideal to be used in combination with other agents that mutually support each other [30]. Indeed, other studies have reported that combinations of ART/ART-related compounds with newer treatment modalities such as erlotinib and rituximab exhibit enhanced activity [66, 67]. The development of strategies for prolonging the effects of sorafenib and other targeting agents is particularly important in metastatic RCC, since tumors typically develop resistance to therapy within 5–11 months. In this study, the proliferation assays presented here revealed that the combination of sorafenib and ART synergistically yielded significantly greater cytotoxicity compared with sorafenib monotherapy alone in RCC cells. Sorafenib is an orally active, multikinase inhibitor that targets the RAF kinases and other receptor tyrosine kinases [VEGFR 1–3, and platelet-derived growth factor receptor-β (PDGFR-β)] [68]. Interestingly, sorafenib-mediated toxicity to leukemia cells has been reported to potentially involve ER stress and ROS generation [77], implying that sorafenib sensitizes RCC cells to ART-mediated oxidative stress. Similarly, *in vivo* anti-tumor and anti-angiogenic efficacy of sorafenib was enhanced in a mouse model of hepatocellular carcinoma by combination with ART without apparent hepatotoxicity [69]. Therefore, combination therapies consisting of anticancer agents that exert oxidative stress together with sorafenib may prove clinically useful for treating RCC, which depends on the dsRNA-activated protein kinase-like ER kinase (PERK) pathway [78].

In summary, ART possesses several characteristics that make it a promising angiogenesis inhibitor for cancer, including its anti-tumor activity, its anti-angiogenic efficacy, its low overall toxicity, and its highly selective toxicity to cancer cells. Given the anti-angiogenic activities described here, ART appears to be well-suited for adjuvant therapy in combination with classical TKIs for the treatment of highly angiogenic RCC. Moreover, ART may be useful for angioprevention, which is a strategy based on nontoxic drugs that can be taken for extended periods or even as life-long treatments to control tumor growth and metastasis. Further *in vivo* studies and clinical trials are necessary to determine whether the promising combination of sorafenib and ART treatment reported here has true therapeutic value.

## MATERIALS AND METHODS

### Clear cell RCC (ccRCC) gene expression and tissue microarray (TMA) datasets

A publicly available dataset consisting of unmatched RNA-sequencing (seq) and clinic-pathologic data from a cohort of 469 patients with ccRCC was obtained from The Cancer Genome Atlas-Kidney Renal Clear Cell Carcinoma (TCGA-KIRC) research network via the TCGA data portal (http://cancergenome.nih.gov/) [70]. In addition, a TMA was generated from surgically removed samples from 119 patients, each of whom had pathologically proven ccRCC, at Samsung Medical Center between August 2005 and October 2010. The study protocol was approved by the institutional review board (IRB) and written informed consent was obtained from all patients prior to sample collection. The patients consisted of 93 males (78%) and 26 females (22%); patient ages ranged from 26–82 years. Of the 119 patients, 67 had stage I (56.3%), 4 patients had stage II (3.4%), 20 patients had stage III (16.8%), and 28 patients had stage IV ccRCC (23.5%). CSS and MFS rates were calculated from the date of histological diagnosis to death and to metastatic recurrence due to ccRCC, respectively. The median follow-up duration was 59 months (interquartile range, 26–70 months).

TMA sections were examined by IHC for TfR1 expression as previously described [71]. Briefly, endogenous peroxidase activity was first blocked by incubating the sections in 0.3% hydrogen peroxide. Antigens were then retrieved by heating the sections in 10 mM sodium citrate (pH 6.0) at 95°C for 30 minutes. Sections were incubated overnight at 4°C with primary rabbit polyclonal antibodies against TfR1 (Abcam) and then for 1 hour at room temperature (RT) with biotinylated secondary antibodies (Vector Laboratories) and a subsequent hour at RT with avidin–biotin complexes (Vector Laboratories). Staining intensity was scored as follows: 0, no staining; 1, weak staining; 2, moderate staining; and 3, strong staining. Before analysis, each sample was classified as having low (none to weak) or high (moderate to strong) TfR1 expression based on the immunoreactivity of TfR1, and the Kaplan-Meier (KM) survival function was plotted. All scoring was performed by a pathologist experienced in evaluating IHC who had no knowledge of the corresponding clinic-pathologic information.

### Cell culture and generation of the SN12C-derived lung metastatic subline SN12C-SRLu2

To study the effect of VHL status on the response to ART, we used Caki-1, 786-O, and SN12C-GFP-tagged cells, all of which are human RCC cell lines. Caki-1 and 786-O cells were obtained from the American Type Culture Collection (ATCC), whereas SN12C-GFP cells were purchased from Metabio (Korea). To exclude the possibility of cross-contamination, all cell lines were authenticated by short tandem repeat (STR) profiling. To generate 786-O-Luc stable cells for *in vivo* bioluminescence imaging (BLI), lentiviral expression particles for firefly luciferase (LVP325, Amsbio) were purchased and immediately used to transduce 786-O cells. Transduced cells were selected by culturing for two weeks in medium containing puromycin (2 μg/mL), and stable clones were assayed for luciferase activity using the Bright-Glo™ luciferase assay (Promega). All cells were maintained in RPMI1640 medium (Gibco) supplemented with 10% FBS (Gibco) and 1% penicillin/streptomycin (PS) and propagated in a 5% CO_2_ atmosphere at 37°C.

To determine whether TfR1 expression is upregulated in human RCC cells with enhanced lung metastatic potential, we isolated lung metastatic cells from the parental SN12C human RCC cell line. To generate the subpopulation of parental SN12C cells capable of lung metastasis in nude mice, we first injected SN12C cells into the subrenal capsules of mice. Orthotopic spontaneous metastatic cells (SN12C-GFP-SRLu1) were then isolated from harvested mouse lungs and expanded, after which they were injected into the subrenal capsules of nude mice. After repeating this injection-isolation-expansion cycle, we obtained the final cell line, designated SN12C-GFP-SRLu2. STR profiling (AmpF/STR Identifiler Kit, Applied Biosystems) was performed to confirm that the parental and metastatic sublines were genetically identical, proving that the metastatic cell line was indeed a derivative of the parental cell line.

### Silencing of TfR1 using transfection with TR1 small interfering RNAs (siRNAs)

For transfection, cells were seeded at a density of 5 × 10^5^ cells/dish and transfected with TfR1-targeting siRNAs or appropriate control siRNA (Santa Cruz Biotechnology), respectively, using Lipofectamine2000 (Invitrogen) according to the manufacturer's protocol. Control and TfR1-targeting siRNAs were purchased from Santa Cruz Biotechnology.

### Compounds

ART was purchased from Selleckchem. A stock solution was prepared by dissolving the drug in dimethyl sulfoxide (DMSO) to a final concentration of 50 mM. The solution was divided into aliquots and stored at −20°C until use.

### Preparation of protein extracts and Western blot analysis

For western blot analysis, cells were lysed with ice-cold RIPA buffer supplemented with a protease inhibitor cocktail and phosphatase inhibitors (Roche Diagnostics). Lysates were clarified by centrifugation at 13000 rpm for 30 min, after which the supernatants were harvested. Protein concentrations were determined using a bicinchoninic acid protein assay kit (Thermo). Equal amounts of protein were subjected to SDS-PAGE and transferred to PVDF membranes (Millipore). After blocking nonspecific binding sites on the membranes with 5% skim milk or 5% BSA for 2 hours at RT, the membranes were incubated with the indicated primary antibodies overnight at 4°C and then with the appropriate secondary antibodies for 1 hour at RT. Antibodies against Cyclin B, Cyclin D1, E2F1, survivin, PARP, EGFR, FAK, Src, α-tubulin, GAPDH (Cell Signaling Technology), TfR1, calpain-1, calpain-2 (Abcam), c-Met (Invitrogen), and c-MYC (Santa Cruz Biotechnology) were obtained commercially. Immunoreactive bands were visualized with HRP-conjugated secondary antibodies and a chemiluminescent substrate by exposure to X-ray film.

### Cell viability assay

RCC cells were harvested upon reaching confluence, seeded into 96-well tissue culture plates at a density of 5 × 10^3^ cells/well, and incubated overnight. The next day, the plating medium was replaced with 0.1 mL fresh culture medium containing vehicle alone as a control, ART, sorafenib, or a combination of sorafenib and ART. Cells were treated with various concentrations of ART in 4-fold and 10-point serial dilution series (*n* = 3 for each condition). After 3 days of incubation at 37°C in a 5% CO^2^ atmosphere, cell viability was assessed using an EZ-Cytox Cell Viability Assay Kit (Daeil Lab). All IC50 values were determined with the S+ Chip Analyzer (Samsung Electro-Mechanics Company, Ltd) and are reported as the averages of three independent experiments. DMSO alone was used as the negative (vehicle) control. Each experiment was carried out in triplicate.

### Cell cycle analysis

Cells were seeded and pretreated with either DMSO (control) or ART (50 μM). After incubation for 48 hours, cells were harvested, washed twice with PBS, and fixed in ice-cold 70% ethanol overnight at 4°C. Cells were fixed in 70% ethanol at 4°C for 30 minutes and then stained with propidium iodide (PI, 40 μg/mL; BD Biosciences) in PBS with RNase (100 μg/mL) at 37°C for 30 minutes. Stained cells were quantified using a fluorescence-activated cell sorter (FACSVerse™, BD). After gating on fluorescence width and area to remove debris and doublet artifacts, the percentages of cells in sub-G1, G1, S, and G2/M phase were determined using the BD FACS software suite.

### Detection of apoptosis by annexin V/PI staining

Cells (1 × 10^6^) were seeded in 60 mm^2^ culture dishes and incubated in a 5% CO_2_ atmosphere at 37°C for 24 hours. Cells were then treated either with ART (50 μM) or DMSO as a negative control. After 48 hours of treatment, adherent and suspension cells were harvested, washed twice with PBS, and resuspended in 1x Annexin V binding buffer (BD). Cells were then incubated with Annexin V-fluorescein-isothiocyanate (FITC) (BD) and PI (Alexa Fluor 488 Annexin V/Dead Cell Apoptosis kit; Life Technologies) according to the manufacturers’ protocols. Apoptosis was subsequently assessed by flow cytometry. Flow cytometry plots were generated with annexin-V on the x-axis and PI on the y-axis and used to determine the percentages of cells in each of the following quadrants: lower left, normal cells; lower right, early apoptotic cells; upper right, late apoptotic and necrotic cells.

### Detection of intracellular ATP

As a readout for Na+/K+-ATPase activity, the intracellular ATP contents of treated cells were measured using a commercial ATP detection kit (ENLITEN^®^ ATP Assay System Bioluminescence Detection Kit for ATP Measurement, Promega) according to the manufacturer's instructions. This kit measures the activity of luciferase on its substrate D-luciferin, which yields a luminescent product. Briefly, cells were washed with PBS and resuspended in lysis buffer. The resultant lysates were mixed with the luciferase/luciferin reagent. The emitted light was immediately quantified using a luminometer and the amounts of ATP were determined by comparison to a standard curve.

### Measurement of ROS

The effect of ART on intracellular ROS production was determined using a ROS Assay Kit (Komabiotech) according to the manufacturer's instructions. In this assay, dichlorodihydrofluorescein diacetate (DCFH-DA), a cell-permeating, non-fluorescent probe, is oxidized by ROS and converted into a fluorescent product, 2′,7′-dichlorofluorescein, which can be measured by flow cytometry. After incubation for 1 hour with DCFH-DA in serum-free medium, the probe was removed and the cells were rinsed with PBS and incubated for 5 hours in fresh medium, either with or without ART (50 μM).

### Electron microscopy

Cell pellets and harvested tumor xenografts were fixed overnight with 2.5% glutaraldehyde in 4% paraformaldehyde at 4°C. After incubation for 1 h in 1% OsO4, the specimens were dehydrated in an ethanol series, passed through propylene oxide, and embedded in epoxy resin (Epok 812, 02-1001, Oken, Japan). Ultrathin sections (60 nm) were collected on 200 mesh nickel grids and stained for 10 min in 1% uranyl acetate and Reynolds’ lead citrate. The specimens were observed with a Hitachi HT7700 electron microscope at 80 kV.

### Clonogenicity assay

Clonogenicity was assessed with a colony-forming assay. Cells were seeded (300 cells/well) in 6-well plates and maintained in complete medium with either DMSO (vehicle) or various concentrations of ART (5, 10, 25 μM) for 2 weeks. After staining with 0.2% crystal violet, the viable colonies in each well were counted.

### *In vitro* transwell invasion assay

Matrigel-coated transwells (BD) were used for the invasion assay. Cells (Caki-1, 2 × 10^5^; 786-O and SN12C-GFP-SRLu2, 2 × 10^5^) that had been serum-starved overnight were suspended in 300 μL serum-free RPMI medium and added to the upper well of a modified Boyden chamber (Corning). Cells were then allowed to invade through the filter, which had been precoated with 100 μg Matrigel (BD Bioscience). Invasion was stimulated by the addition of complete medium supplemented with 10% FBS, either with or without ART (5, 10, 25 and 50 μM), to the bottom compartment. Cells were allowed to invade through the Matrigel-coated filter for 24 hours, after which the cells that had invaded the bottom chamber were counted. The upper side of the membrane was scraped with a cotton swab to remove cells that had attached but not invaded, and the invading cells attached to the lower membrane surface were fixed with 4% paraformaldehyde (PFA) and stained with H&E. The degree of invasion was assessed in each well by counting the number of cells in 10 randomized fields at x100 magnification. Using the cell viability assay to correct for the proliferative effects of 10% FBS, the degree of invasion was adjusted using the formula (corrected invasion index = number of invaded cells/percentage of viable cells) [72].

### Endothelial cell culture

HUVECs were obtained from the American Type Culture Collection (ATCC, Rockville, MD, USA) and grown in the presence of endothelial cell growth medium (EGM-2, Lonza) on plates coated with 2% gelatin. Medium was supplemented with 1% P/S, and cells were grown at 37°C in a 5% CO_2_ atmosphere.

### Endothelial cell proliferation assay

HUVECs were seeded in 96-well plates (2 × 10^4^ cells/well). After 24 hours of incubation, cells were incubated with various concentrations of ART for 72 hours and then with EZ-CYTOX solution for 4 hours. The absorbance of each well at 540 nm was determined using an ELISA reader (Bio Tek Instruments, Burlington, VT, USA). All results are expressed as IC50 values.

### Endothelial cell transwell migration assay and wound healing assay

Endothelial cell migration was analyzed using 0.1% gelatin-coated 24-well transwell inserts with a pore size of 8 μm (Corning Costar Corp). HUVEC cells were plated on the upper chamber in serum-free medium with either DMSO (control) or various concentrations of ART. The lower chamber contained 10% FBS as a chemoattractant. Cells were incubated at 37°C in the presence of CO_2_ for 4 hours, after which the cells that had migrated to the lower membrane surface were fixed with cold 4% PFA and stained with H&E. Images of invaded cells were captured at ×100 magnification (three fields per well), and the numbers of invaded cells were determined using ImageJ software.

For the *in vitro* wound healing assay, HUVEC cells (2 × 10^5^) were seeded in 6-well plates and grown overnight. After cells had completely adhered to the plates, 3 vertical lines were scraped in each monolayer (9 crosses per plate) using a yellow pipette tip. After scraping, cells were washed with PBS and then incubated with VEGF-containing medium, either with or without various concentrations of ART. Images of each cross were obtained at different time intervals (magnification 100×) using a microscope video system. The widths of the four sides in each cross were measured and then averaged. Migratory ability was calculated using the following formula: 100% − (width 24 hours/width 0 hours) × 100%.

### Endothelial cell tube formation assay

The tube formation assay, which assesses angiogenesis, was conducted to determine whether ART exerts an anti-angiogenic effect. Briefly, 96-well plates were preincubated with growth factor reduced Matrigel (Corning) at 37°C for 30 minutes. After overnight pretreatment with ART (50 μM), cells were harvested and resuspended in EBM medium supplemented with 50 ng/ml VEGF and either DMSO (control) or ART (5, 10, 25, 50 μM). Cells were then seeded into the Matrigel-coated wells. After incubation at 37°C in the presence of 5% CO_2_ for 4 hours, tube formation was observed using a light microscope and images were captured. The total lengths of the tubular structures in randomly chosen microscope fields (three per well) were measured using ImageJ. HUVECs that were plated on the Matrigel surface successfully formed tubular structures; the lengths of these structures in the presence or absence of ART were all measured within 6 hours.

### Western blot analysis of lysates from HUVECs

To determine the effects of ART on VEGFR2-dependent signaling cascades, HUVECs were serum-starved and incubated with ART overnight, after which they were treated with VEGF (50 ng/mL) for 10 min. Cells were lysed with ice-cold RIPA buffer containing a protease inhibitor cocktail and phosphatase inhibitors (Roche). Protein concentrations were determined using a modified BSA assay kit (Thermo) and normalized before loading on 10% SDS-PAGE gels. After protein transfer to membranes, the membranes were probed with specific antibodies (Cell Signaling Technology) against VEGFR2, phospho-VEGFR2 (Tyr1175), Erk1/2, phospho-Erk1/2 (Thr202/Tyr204), FAK, phospho-FAK (Tyr397), Src, phospho-Src (Tyr416), AKT, phospho-AKT (Ser473), NF-κB p65, and phospho-NF-κB p65 (Ser536). Membranes were then incubated with HRP-conjugated secondary antibodies and immunoreactive bands were visualized with a chemiluminescent substrate via exposure to X-ray film.

### *In vivo* tumorigenicity assay using bioluminescence imaging

All animals received humane care in compliance with the “Guide for the Care and Use of Laboratory Animals” prepared by the Institute of Laboratory Animal Resources (National Institutes of Health). Additionally, all animal procedures were performed according to the Animal Experiment Guidelines of Samsung Biomedical Research Institute. For the subcutaneous delivery model, 6–8-week-old female BALB/c nude mice were obtained from Orient Bio (Korea). The effects of ART on the tumorigenic potential of RCC cells were analyzed via subcutaneous injection of 786-O-Luc cells [5 × 10^6^ in 200 μL Hank's Balanced Salt Solution (HBSS)] after administration of anesthesia. When the tumors reached an average volume of 100 mm^3^, 13 nude mice were randomly divided into two groups and treatment with ART or vehicle (as a control) was initiated. Six of the nude mice were treated with ART diluted in sterile saline with 5% sodium bicarbonate, which was administered once daily by intraperitoneal injection (100 mg/kg). The remaining seven nude mice in the control group were administered sterile saline according to a timing and dosing schedule identical to that used for the treated group.

To assess the growth of the subcutaneously implanted 786-O-Luc tumor cells, *in vivo* imaging was used to quantify the bioluminescence signals. To this end, mice were anesthetized with isoflurane and intravenously administered a D-luciferin solution (*in vivo* imaging solutions, PerkinElmer, 150 mg/kg in PBS). Images were acquired with an IVIS Spectrum imaging system (PerkinElmer) 2–5 min after injection, and the bioluminescence signal on each image was quantified using the Living Image Software package (PerkinElmer/Caliper Life Sciences). Briefly, the photon flux (photons/s/cm^2^/steradian) was measured within a region of interest (ROI) drawn around each subcutaneous tumor. At the end of the experiment, mice were sacrificed using a standard method. Tumors were removed and fixed in 10% buffered formalin before paraffin processing. The tumor specimens were sectioned (4 μm), with the largest cross sections corresponding to the ultrasound imaging planes. The sections were stained with H&E and assessed microscopically for changes in cell morphology.

### Human RCC pulmonary metastasis assay

For *in vivo* lung metastasis experiments, 1 × 10^6^ of 786-O-Luc cells were intravenously inoculated into the lateral tail vein of female 6- to 8-week-old BALB/c-nu mice. One days after injection, mice were randomly divided into two groups (10 mice per group), and ART (100 mg/kg) or vehicle (as a control) was administered once daily by intraperitoneal injection. The mice were killed after 3 weeks of injection, and harvested lungs were processed for paraffin-embedded sections, which were stained with H&E to confirm pathologically the presence of lung metastatic tumors.

### *In vivo* immunohistochemistry

The proliferation index in 786-O xenografts was determined by staining tumor sections with anti-Ki-67 antibodies (Zymed Laboratories) at a 1:50 dilution. The extent of apoptosis in each tumor was measured by TUNEL using the TdT-Fragel DNA Fragmentation Detection Kit (Calbiochem) according to the manufacturer's protocol. All slides were also counterstained with Mayer's hematoxylin. Endothelial cells were immunostained using rat monoclonal anti-CD34 antibodies at a 1:50 dilution (Abcam). The levels of phospho-ERK1/2 were determined using anti-pERK1/2 antibodies (phospho-p44/42; Cell Signaling Technology) at a 1:100 dilution.

### Statistical analysis

CSS and MFS values were calculated by the Kaplan–Meier method and compared with the log-rank test. Group comparisons were performed using Student's *t*-test (two-tailed). Differences with *P* values less than 0.05 were considered statistically significant.
